# LncRNA PTOV1-AS2 Promotes Colon Cancer Progression through the miR-145-5p/FSCN1 Axis

**DOI:** 10.1155/2023/1298312

**Published:** 2023-03-14

**Authors:** Zhen Zhang, Honglei Wu, Zhaosheng Chen, Guangchun Li, Jianqiang Guo

**Affiliations:** The Second Hospital of Shandong University, Jinan 250033, China

## Abstract

**Objective:**

The long noncoding RNA (lncRNA) gene PTOV1-AS2 is a potentially oncogenic lncRNA gene. However, its role and regulatory mechanism in the occurrence and development of colon cancer are still unclear. In this study, the lncRNA PTOV1-AS2 was used as a starting point to investigate the role of competing endogenous RNA (ceRNA) regulatory mechanisms in colon cancer.

**Methods:**

The expression of lncRNA PTOV1-AS2 mRNA in colon cancer tissues and cell lines was measured by quantitative real-time polymerase chain reaction (qRT-PCR) and screened for differential expression in cells. We examined the effects of lncRNA PTOV1-AS2 overexpression or downregulation of its expression on various cellular processes in HCT116 and SW620 cells after the transfection with an overexpression construct or PTOV1-AS2p-specific shRNA, respectively. In particular, we examined the effects on cell proliferation, migration, and invasion using the cell counting kit-8 CCK-8 assay and Transwell migration and invasion assays, respectively. In addition, the binding targets of lncRNA PTOV1-AS2/miR-145-5p and miR-145-5p/FSCN1 were predicted using various bioinformatics tools and validated by a dual luciferase assay. We also examined the effect of the lncRNA PTOV1-AS2/miR-145-5p axis on FSCN1 expression by qRT-PCR analysis. Furthermore, we investigated the effect of the PTOV1-AS2/miR-145-5p/FSCN1 axis on the biological function of colon cancer cells using an *in vitro* colon cancer cell model with reduced expression of PTOV1-AS2 and simultaneous transfection of a miR-145-5p inhibitor or FSCN1 vector. Additionally, we established a colon cancer xenograft tumor nude mouse model and used it to investigate the effect of locally injected lncRNA PTOV1-AS2 vector on the tumor growth and survival status of tumor-bearing mice.

**Results:**

We found that PTOV1-AS2 was highly expressed in colon cancer, which was associated with worse survival. High expression of PTOV1-AS2 promoted cell proliferation, migration, and invasion, while low expression of PTOV1-AS2 inhibited these processes in HCT116 and SW620 cells. The microRNA miR-145-5p was found to bind to the 3′-UTR region of both PTOV1-AS2 and FSCN1. In addition, miR-145-5p decreased the protein expression of its target gene FSCN1 and reduced the PTOV1-AS2-induced expression of FSCN1 in colon cancer cell lines. Also, silencing miR-145-5p or enhancing FSCN1 expression could partially restore the inhibition of cell proliferation, migration, invasion, and the tumorigenic capacity caused by silencing the expression of PTOV1-AS2 *in vitro* and *in vivo*.

**Conclusion:**

PTOV1-AS2 promotes colon cancer progression by “sponging” miR-145-5p to upregulate FSCN1.

## 1. Introduction

Colon cancer is the third most common cancer worldwide and the second leading cause of cancer-related deaths, whose risk factors include unhealthy diet, obesity, sedentary lifestyle, and lack of regular screenings. The incidence and mortality rates of colon cancer vary widely across the world. Specifically, colon cancer incidence rates are highest in Europe, Australia/New Zealand, and North America and lower in most of Africa and South-Central Asia [1]. Colon cancer incidence and mortality rates in highly developed countries are among the highest rates globally, and although they have shown a stable and decreasing trend in recent years, they are rapidly increasing in many low and middle-income countries [2]. The incidence and mortality of early-onset colon cancer (usually defined as colon cancer diagnosed before the age of 50 years) have recently been on the rise globally [3, 4]. Despite great advances in the diagnosis and treatment of colon cancer in recent years, the prognosis of many patients remains poor due to the lack of timely diagnosis and personalized disease management strategies. Most patients are diagnosed at advanced stages with metastases, which limits the treatment options [5]. Increased research on diagnostic predictive markers and prognostic features of specific treatment regimens for colon cancer can help in patient screening, adjuvant therapy, and recurrence and metastasis prevention. Among them, biomarkers are less invasive and allow for more accurate prediction of disease onset, progression, and recurrence.

Currently, lncRNAs have shown potential value as diagnostic and prognostic markers in various cancer studies. LncRNAs are transcripts longer than 200 nucleotides that generally lack the potential to be translated into proteins [6]. Although lncRNAs do not encode proteins, they have been shown to regulate a variety of cellular processes, such as DNA replication and repair, chromatin remodeling, genome structure, transcription, mRNA splicing, translation, RNA stabilization, transcriptional regulation, and signaling pathways [7, 8]. Dysregulation of lncRNAs has an important role in cancer development and progression and may influence tumorigenesis by acting as oncogenes [9]. For example, LncRNA MIR31HG is highly expressed in lung adenocarcinoma tissues and cells, promotes cancer cell proliferation, and serves as a prognostic biomarker for lung adenocarcinoma [10]. Also, overexpression of lncRNA MEG3 increases the expression of p53 and reduces the proliferation and metastasis of gastric cancer cells [11].

LncRNAs regulate the expression level of target genes by sponging endogenous miRNAs, thereby inhibiting the repressive effect of miRNAs on target genes [12]. In general, miRNAs are small noncoding RNAs of approximately 21–23 nucleotides in length and have critical roles in embryonic development, tissue differentiation, cell differentiation, cell cycle regulation, and apoptosis [13]. Dysregulation of miRNAs has far-reaching effects on the development and progression of human cancers. In particular, miRNAs have roles in promoting tumor invasion, growth, immune invasion, and angiogenesis but can also suppress tumor formation, growth, and metastasis [14]. This is attributed to the targeting of different genes by miRNAs, and dysregulated miRNAs may act as tumor suppressor genes or oncogenes in tumors [15]. Oncogenic miRNAs are overexpressed in tumors and promote cell cycle progression in cancer cells, while oncogenic miRNAs are lowly expressed in tumors, and their high expression induces cell cycle arrest in cancer cells [16]. The lncRNA-miRNA axis may exert both tumor-suppressive and oncogenic effects through specific interactions between lncRNAs and miRNAs, implying that the lncRNA-miRNA axis is crucial for targeting.

A microRNA, miR-145-5p, has been shown to play a role in several diseases. It is encoded by the MIR145 gene on chromosome 5. It has been mainly implicated as a tumour suppressor miRNA in various cancers. Few studies, however, have reported upregulation of this miRNA in some cancers [17]. As a muscle-binding protein, FSCN1 plays a key role in cell migration, motility, adhesion, and cell-cell interactions. Furthermore, some studies have found that FSCN1 is aberrantly expressed in various cancers and is closely associated with the malignant behaviour of tumours [18].

This study aimed to determine the role of lncRNA PTOV1-AS2 in the progression of colon cancer. We also explored the interaction between lncRNA PTOV1-AS2 and miR-145-5p and its target gene FSCN1 in order to determine the prospective application of the lncRNA PTOV1-AS2/miR-145-5p/FSCN1 axis in the diagnosis and prognosis of colon cancer.

## 2. Materials and Methods

### 2.1. Clinical Samples

The population in this study consisted of colon cancer patients who underwent surgical treatment at The Second Hospital of Shandong University between February 2019 and October 2021. All colon cancer patients included in this study had pathologically and histologically confirmed diagnoses, and none of these patients had received treatment, such as chemotherapy and/or radiotherapy before surgery. Before surgery, all patients signed an informed consent form for scientific research. This study was approved by the Medical Ethics Committee of The Second Hospital of Shandong University. Tissue specimens were sampled during surgery, snap-frozen in liquid nitrogen immediately after sampling, and transferred to a −80°C cryogenic freezer for storage after snap-freezing overnight. Forty-five pairs of colon cancer tissue samples and paired distant cancer normal colorectal tissue samples were randomly selected.

### 2.2. Cell Culture and Transfection

A normal colon epithelial cell line (HIEC-6) and four colon cancer cell lines (SW480, SW620, HCT116, and HT29) were purchased from the National Collection of Authenticated Cell Cultures (Shanghai, China).

All cells were cultured in Dulbecco's modified Eagle's medium (DMEM; Gibco/Thermo Fisher Scientific, Inc., Waltham, MA, USA) and maintained in an incubator at 37°C, with 5% CO2 and 95% humidity.

The miR-145-5p mimics (sense: 5′-GUCCAGUUUUCCCAGGAAUCCCU-3′, antisense: 5′-GGAUUCCUGGGAAAACUGGACUU-3′) and miR-145-5p inhibitors (5 5′-AGGGAUUCCUGGGAAAACUGGAC-3′) [19] were purchased from BGI Genomics Co., Ltd. (Shenzhen, China). The PTOV1-AS2 overexpression plasmid was constructed as follows: the PTOV1-AS2 gene sequence was obtained from the NCBI website, and then the linear cDNA sequence was synthesized manually, subcloned into the pcDNA3.1 vector and verified by one-generation DNA sequencing. The PTOV1-AS2 silencing vector was constructed as follows: the interference target sequence was designed based on the PTOV1-AS2 gene sequence, subcloned into the pLKO.1 lentiviral eukaryotic expression vector and verified by one-generation DNA sequencing. The constructs were transfected or combinatorially transfected into colorectal cancer cell lines SW620 and HCT116, respectively. After 48 h of transfection, cells were used in various experiments for gene expression analysis by quantitative real-time polymerase chain reaction (qRT-PCR), cell proliferation by cell counting kit-8 (CCK-8) assay, and cell migration and invasion by Transwell assays.

### 2.3. Measurement of Proliferation by the CCK-8 Assay

The transfected cells of each group and cells of the corresponding control group were cultured for 0, 24, 48, 72, and 96 h and subjected to the proliferation assay by adding 10 *μ*L CCK-8 solution at each time point and continuing to culture for 2 h. Eventually, the absorbance (A) value at 450 nm was measured, and the cell proliferation ability was statistically analyzed.

### 2.4. Detection of Cell Migration and Invasion by Transwell Assay

During the invasion assay, the upper (luminal) surface of the bottom membrane was covered with 50 mg/L Matrigel (1 : 8). After digestion of the cells and discarding the medium, the cell density was adjusted to 4 × 10^5^ cells/mL with serum-free medium containing bovine serum albumin. The lower chamber was filled with 600 *μ*l of complete medium containing 10% fetal bovine serum (FBS), and after 24 h, the medium was removed from the lower chamber. Then, after fixing the cells with a 90% ethanol solution (v/v) for 30 min at room temperature, the cells were air-dried and stained with 0.1% crystal violet solution at room temperature for 10 min. Eventually, the cells in the upper chamber were wiped off with a cotton swab, and the number of cells in the lower chamber were counted in five randomly observed fields of view. As for the migration assay, no stromal gel was used and the rest of the experimental steps were the same as those of the invasion assay.

### 2.5. Gene Expression Analysis by qRT-PCR

Gene expression analysis was performed according to the protocols provided by the manufacturers of the qRT-PCR reagents and kits. The PCR reaction conditions were as follows: (1) predenaturation at 95°C for 1 min; (2) 40 cycles of denaturation at 95°C for 15 s, annealing at 60°C for 60 s, and extension at 72°C for 45 s. Ultimately, the ratio of gene expression in the experimental and control groups was calculated according to the following equation: folds = 2^−[*Ct*(PTOV1-AS2 or FSCN1)−*Ct*(GAPDH)] experimental group−[*Ct*(PTOV1-AS2 or FSCN1)−*Ct*(GAPDH)] control group^. GADPH was used as the internal reference. The forward primer for PTOV1-AS2 was 5′-CGGCACTAGGGAAACGTCAT-3′, and the reverse primer was 5′-TGTCCACCGATGATCTCCCT-3′; the forward primer for FSCN1 was 5′-CCAGGGTATGGACCTGTCTG-3′, and the reverse primer was 5′-GGTGTGGGTACGGAAGGCAC-3′ and the forward primer for GAPDH was 5′-GACAGTCAGCCGCATCTTCT-3′, and the reverse primer was 5′-GCGCCCAATACGACCAAATC-3′.

### 2.6. Luciferase Reporter Assay

Wild-type or mutant 3′ UTR of FSCN1 and PTOV1-AS2 was cloned into the downstream of luciferase reporter gene of pmiR-GLO vectors. Then, the recombinant reporter gene vectors were transfected into cells with miR-145-5p mimic or NC mimic. Cells were cultured for 48 h. The luciferase activity was determined using the dual-luciferase reporter gene system.

### 2.7. Western Blot Analysis of FSCN1 Protein Expression

Whole cells or tissues were lysed in a lysis buffer, and protein concentration was determined using the Pierce bicinchoninic acid (BCA) Protein Assay Kit (Pierce Biotechnology, Rockford, IL, USA). Protein samples were separated by electrophoresis on a 10% polyacrylamide sodium dodecyl sulfate (SDS) gels and then transferred to polyvinylidene fluoride (PVDF) membranes (MilliporeSigma, Burlington, MA, USA). Membranes were incubated at 4°C overnight with a primary anti-FSCN1 antibody (1 : 5,000 dilution), followed by incubation at room temperature for 1 h with the appropriate HRP-labelled secondary antibody (1 : 500 dilution). The immunoreactive band signal intensity was visualized using enhanced chemiluminescence. Separate blots were used for each individual experiment to avoid artifacts associated with incomplete antibody stripping.

### 2.8. *In Vivo* Validation of the Effects of the PTOV1-AS2/miR-145-5p/FSCN1 Axis on Colorectal Cancer Growth in a Nude Mouse Tumor Model

All animal experiments were approved by the Medical Ethics Committee of The Second Hospital of Shandong University and conducted in accordance with the Animal Protection Law of the People's Republic of China. The 4-6-week-old BALB/c nude mice used in this study were purchased from Vital River Laboratories (Vital River, Beijing, China). Different groups of colon cancer stable transitional cell lines were cultured to logarithmic growth phase, trypsin digested, centrifuged at 5,000 rpm/min for 5 min, and washed three times with cold phosphate-buffered saline (PBS). Then, after resuspending cells in PBS, cell suspensions were prepared by mixing the resuspended cells with Matrigel in a 5 : 1 ratio and used as inoculum suspensions for individual nude mice. Ultimately, 120 *μ*L of the cell suspension was injected subcutaneously into the posterior coeliac of nude mice. The width and length of tumors were measured every 7 days, and the tumor volume was calculated. The tumor-bearing mice were observed for survival status and the tumor growth.

### 2.9. Immunohistochemistry Analysis

Tissue sections underwent xylene deparaffinization and ethanol rehydration. Antigen retrieval was conducted for 30 min by citrate buffer boiling (pH 6.0, 10 mM). After the inhibition of endogenous peroxidase activity (10 min, 0.3% H_2_O_2_), sections experienced blocking in 2% serum in PBS and FSCN1-antibody incubation. The protein semiquantitative expression was determined by the integrated option density (IOD) of FSCN1.

### 2.10. Statistical Analysis

The SPSS software (IBM Corporation, Armonk, NY, USA) and GraphPad Prism 6 software (GraphPad Software Inc., San Diego, CA, USA) were used to analyze all data. For *in vitro* and *in vivo* experiments, differences between groups were assessed using *t*-tests or analysis of variance (ANOVA) tests. All *P* values were two-tailed, and *P* < 0.05 was considered statistically significant. All data are expressed as the mean ± standard deviation (SD) of at least three independent replicates.

## 3. Results

### 3.1. PTOV1-AS2 is Highly Expressed in Colon Cancer and Is Associated with Worse Survival

**Figure 1:**
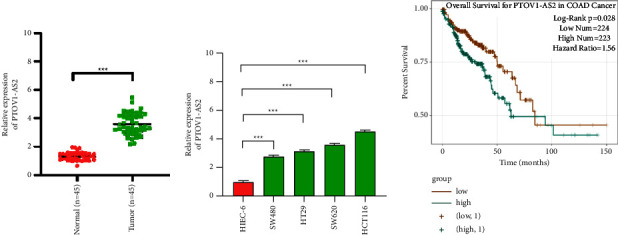
PTOV1-AS2 is highly expressed in colon cancer, which is associated with worse survival. (a) Measurement of PTOV1-AS2 expression in colon cancer tissues (*n* = 45) and paracancerous tissues (*n* = 45) by qRT-PCR analysis. (b) Measurement of PTOV1-AS2 expression in normal intestinal epithelial cells HIEC-6 and colon cancer cell lines SW480, HT29, SW620, and HCT116 by qRT-PCR analysis. (c) Survival curves of colon cancer patients with different PTOV1-AS2 expression levels were plotted based on results obtained by bioinformatics analysis using the ENCORI database (https://starbase.sysu.edu.cn/). ^*∗∗∗*^*P* < 0.001.

We measured the expression level of PTOV1-AS2 in cancerous and paracancerous specimens collected from colon cancer patients, and the results revealed that the expression level of PTOV1-AS2 in cancer tissues was higher than that in paracancerous tissues (Figure 1(a)). In addition, the expression of PTOV1-AS2 was also found to be increased in four colon cancer cell lines, with the highest expression found in HCT116 and SW620 cells (Figure 1(b)). Also, based on the analysis of the encyclopedia of RNA interactomes (ENCORI) database, the survival curves suggested that the postoperative survival of colon cancer patients with high PTOV1-AS2 expression was clearly shorter than that of patients with low PTOV1-AS2 expression (*P*=0.028, Figure 1(c)). We thus hypothesize that PTOV1-AS2 may play a certain role in colon cancer.

### 3.2. PTOV1-AS2 Promotes the Proliferation, Migration, and Invasion Abilities of Colon Cancer Cells

**Figure 2:**
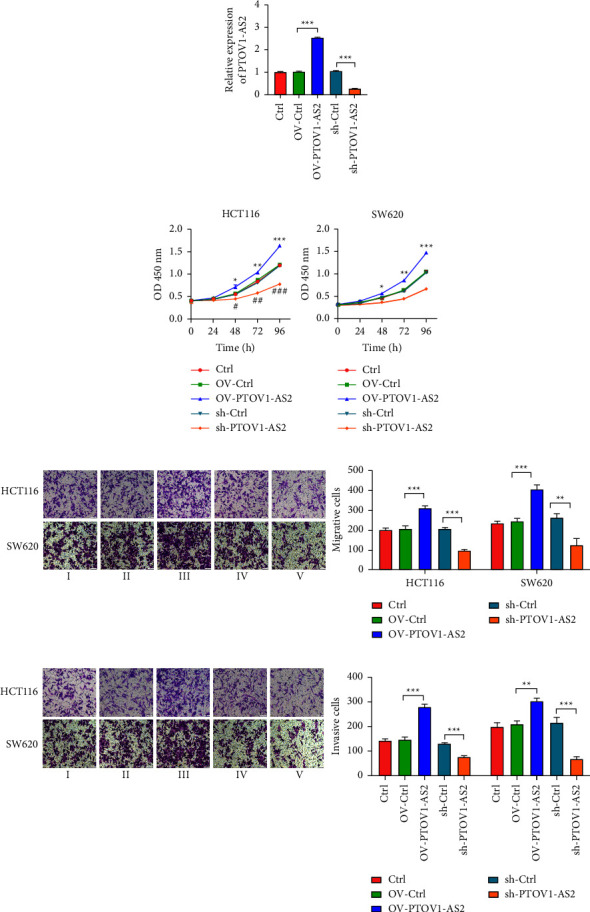
Effects of PTOV1-AS2 on the proliferation, migration, and invasion abilities of colon cancer cells. (a) PTOV1-AS2 overexpression efficiency and its reduction in expression in colon cancer cells were evaluated by qRT-PCR analysis. ^*∗*^: vs. OV-Ctrl, ^#^: vs. sh-Ctrl. (b) Cell proliferation ability was assessed by the CCK-8 assay. (c, d) Transwell assays were used to assess the migration (c) and invasion (d) abilities of cells. ^*∗*^*P* < 0.05, ^*∗∗*^*P* < 0.01, ^*∗∗∗*^*P* < 0.001, ^#^*P* < 0.05, ^##^*P* < 0.01, ^###^*P* < 0.001.

To explore the potential roles played by PTOV1-AS2 in colon cancer, we overexpressed PTOV1-AS2 by transfection with an overexpression plasmid or reduced its expression by transfection with a PTOV1-AS2p-specific shRNA in HCT116 and SW620 cells. After confirming its overexpression efficiency or reduction in expression by qRT-PCR analysis (Figure 2(a)), we assessed the proliferation ability of cells transfected with these constructs using the CCK-8 assay. The results showed that overexpression of PTOV1-AS2 promoted cell proliferation and reduced expression of PTOV1-AS2 inhibited cell proliferation in the two colon cancer cell lines that were tested (Figure 2(b)). Similarly, the Transwell assay results showed that the migration and invasion abilities of the cells were enhanced after overexpression of PTOV1-AS2 but reduced after knockdown of PTOV1-AS2 in HCT116 and SW620 cells (Figures 2(c) and 2(d)). These results suggest that PTOV1-AS2 promotes the cell proliferation, migration, and invasion of colon cancer cells.

### 3.3. Highly Expressed PTOV1-AS2 Competitively Binds miR-145-5p and Enhances FSCN1 Expression in Colon Cancer Cell Lines

**Figure 3:**
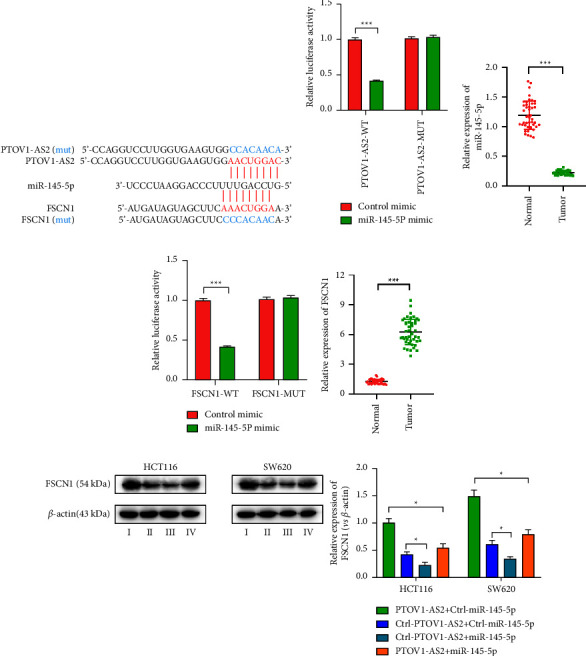
PTOV1-AS2/miR-145-5p axis affects the expression of FSCN1 (a) Bioinformatics analysis predicted that miR-145-5p can bind the 3′-UTR region of both PTOV1-AS2 and FSCN1. (b) Dual luciferase assay confirmed the target binding of PTOV1-AS2 and miR-145-5p. (c, e) Measurement of miR-145-5p (c) and FSCN1 (e) expression in colon cancer tissues by qRT-PCR analysis. (d) Dual luciferase assay to verify the targeted binding of miR-145-5p to the FSCN1 3′-UTR region. (f) Analysis of the regulation of the protein expression levels of FSCN1 by PTOV1-AS2/miR-145-5p axis by western blot analysis. I: PTOV1-AS2 + Ctrl-miR-145-5p; II: Ctrl-PTOV1-AS2 + Ctrl-miR-145-5p; III: Ctrl-PTOV1-AS2 + miR-145-5p; IV: PTOV1-AS2 + miR-145-5p. ^*∗*^*P* < 0.05, ^*∗∗∗*^*P* < 0.001.

LncRNAs may act as competing endogenous (ceRNAs) to sponge miRNAs and regulate the expression of miRNA target genes, thereby playing a critical role in tumorigenesis. Thus, we used a combination of bioinformatic-based prediction approaches using the ENCORI (https://starbase.sysu.edu.cn/), AnnoLnc2 (https://annolnc.gao-lab.org/), and miRDB (https://mirdb.org/) databases to predict that PTOV1-AS2 binds miR-145-5p (Figure 3(a)), and such binding was confirmed by dual luciferase assay (Figure 3(b)). The results of the qRT-PCR analysis showed that miR-145-5p was lowly expressed in colon cancer tissues (Figure 3(c)). Furthermore, analysis using the miRDB (https://mirdb.org/), TargetScan (https://www.targetscan.org/vert_72/), and miRBase (https://www.mirbase.org/) databases predicted that miR-145-5p binds the FSCN1 3′-UTR region (Figure 3(a)), and such binding was also confirmed by dual luciferase assay (Figure 3(d)). The results of the qRT-PCR analysis showed that FSCN1 was highly expressed in colon cancer tissues (Figure 3(e)). In addition, the results of the analyses of a colon cancer cell line model, simultaneously transfected with PTOV1-AS2 overexpression and miR-145-5p mimic plasmids, revealed that miR-145-5p downregulated the protein expression of the target gene FSCN1 and reduced the PTOV1-AS2-induced expression of FSCN1 (Figure 3(f)). These results imply that PTOV1-AS2 may upregulate FSCN1 expression by competitively binding miR-145-5p in colon cancer cell lines.

### 3.4. PTOV1-AS2 Exerts Oncogenic Effects in Colon Cancer Cells through a ceRNA Mechanism

**Figure 4:**
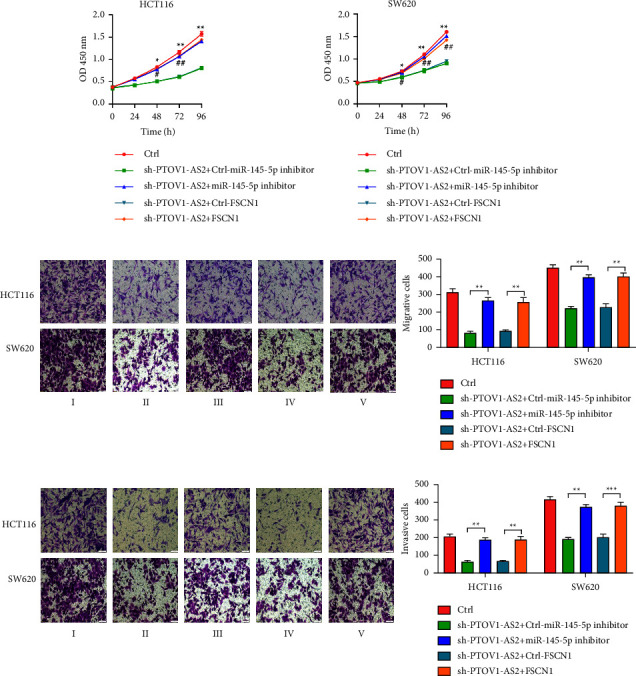
PTOV1-AS2/miR-145-5p/FSCN1 axis regulates biological functions of colon cancer cells (a) Cell proliferation ability was assessed by the CCK-8 assay. ^*∗*^: I vs. II, ^#^: III vs. IV. (b, c) Transwell assays were used to assess the migration (b) and invasion (c) abilities of colon cancer cells. I Ctrl; II: sh-PTOV1-AS2 + Ctrl-miR-145-5p inhibitor; III: sh-PTOV1-AS2 + miR-145-5p inhibitor; IV: sh-PTOV1-AS2 + Ctrl-FSCN1; V sh-PTOV1-AS2 + FSCN1. ^*∗*^*P* < 0.05, ^*∗∗*^*P* < 0.01, ^*∗∗∗*^*P* < 0.001, ^#^*P* < 0.05, ^##^*P* < 0.01, ^###^*P* < 0.001.

We also examined the effect of the PTOV1-AS2/miR-145-5p/FSCN1 axis on the biological functions of colon cancer cells using a colon cancer cell line model with silenced expression PTOV1-AS2 and simultaneous silencing of miR-145-5p or upregulated FSCN1 expression. To this end, we investigated the changes in the proliferation, migration, and invasion abilities of these cells using the following experimental groups: Ctrl, sh-PTOV1-AS2 + Ctrl-miR-145-5p inhibitor, sh-PTOV1-AS2 + miR-145-5p inhibitor, sh-PTOV1-AS2 + Ctrl-FSCN1, and sh-PTOV1-AS2 + FSCN1. The results showed that silencing miR-145-5p or upregulating FSCN1 expression could partially restore the reduced cell proliferation (Figure 4(a)), migration (Figure 4(b)), and invasion (Figure 4(c)) caused by silencing the expression of PTOV1-AS2 in the two colon cancer cell lines.

### 3.5. Confirmation of the Regulatory Role of the PTOV1-AS2/miR-145-5p/FSCN1 Axis in Colon Cancer Growth in an *in Vivo* Nude Mouse Tumor Model

**Figure 5:**
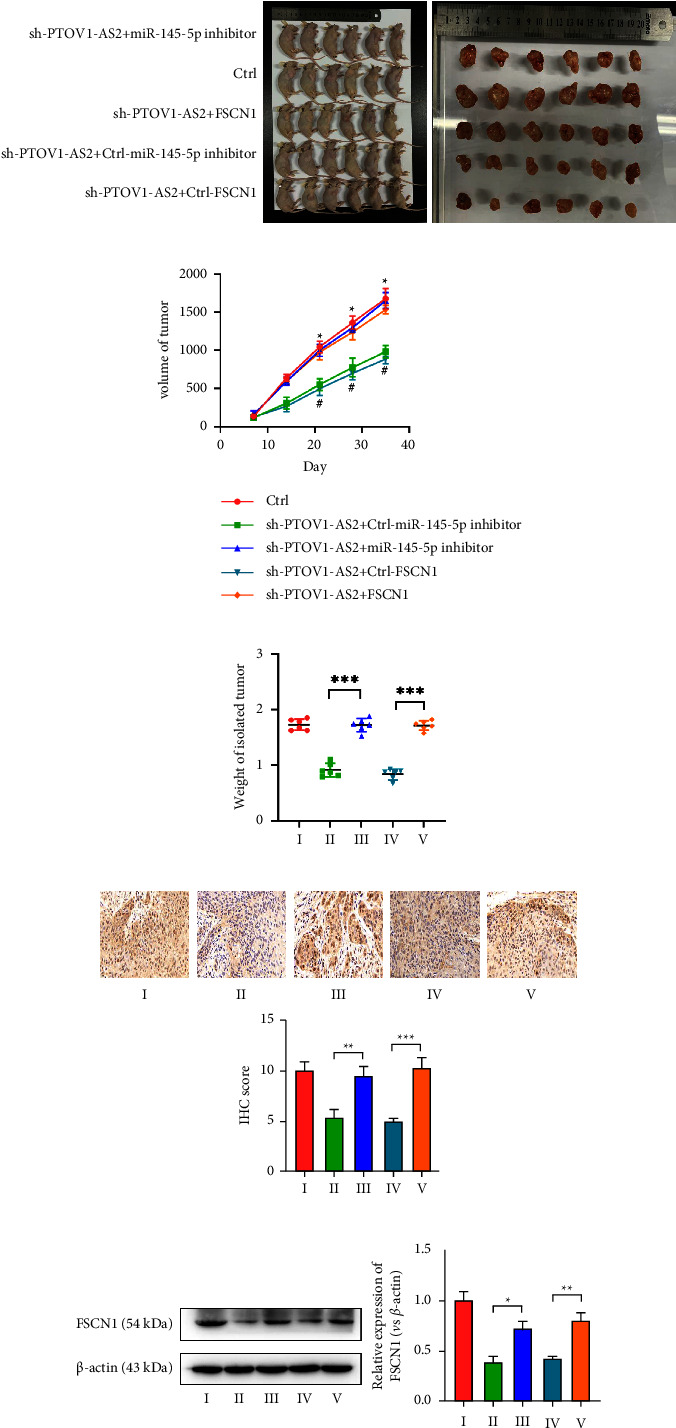
Confirmation of the regulatory role of the PTOV1-AS2/miR-145-5p/FSCN1 axis in the colon cancer growth *in vivo* (a) Images of mice and tumors in the nude mouse tumor model. (b) Volume of the isolated tumors. (c) Weight of isolated tumors. (d) Detection of Ki67 expression in tumors by immunohistochemical staining. (e) Western blot analysis of FSCN1 protein expression in tumors. I Ctrl; II: sh-PTOV1-AS2 + Ctrl-miR-145-5p inhibitor; III: sh-PTOV1-AS2 + miR-145-5p inhibitor; IV: sh-PTOV1-AS2 + Ctrl-FSCN1; V sh-PTOV1-AS2 + FSCN1. ^*∗*^*P* < 0.05, ^*∗∗*^*P* < 0.01, ^*∗∗∗*^*P* < 0.001.

In accordance with the results of cellular experiments, we verified the role of the PTOV1-AS2/miR-145-5p/FSCN1 axis in an *in vivo* nude mouse tumor model. Nude mice were sacrificed at the end of week 7, photographed (Figure 5(a)), and weighed to determine the tumor weight. The results showed that silencing the expression of PTOV1-AS2 reduced the tumorigenic capacity of colon cancer cells, as evidenced by lower tumor weight (Figure 5(b)) and volume (Figure 5(c)). Silencing miR-145-5p or enhancing FSCN1 expression could partially reduce the tumorigenic capacity caused by silencing the expression of PTOV1-AS2. The results of the measurement of the expression of the proliferation-associated nuclear antigen Ki67 were consistent with its tumorigenic ability (Figure 5(d)). Furthermore, the analysis of FSCN1 expression in isolated tumors showed that PTOV1-AS2 enhanced FSCN1 expression by competitively binding miR-145-5p (Figure 5(e)).

## 4. Discussion

Due to changes in diet and lifestyle, as well as inadequate coverage of screening and effective treatment, the incidence and mortality of colon cancer are increasing, ranking third in incidence and second in mortality among all cancers worldwide. Since the clinical symptoms of colon cancer during early stages are not obvious, most patients only seek treatment when it is clinically advanced, and as a result, its survival rate is lower compared with other cancers. Early detection is an important determinant in preventing colon cancer metastasis, reducing mortality, and improving prognosis and future quality of life. Traditional prognostic factors for malignancy include clinical and pathological criteria, such as the patient's age, tumor size and tumor grade, number of affected local lymph nodes, and the presence and grade of metastases. Although these factors have been used for decades, they present serious limitations in predicting patient prognosis [20]. The identification of robust prognostic and/or predictive biomarkers for patients with colon cancer is essential to develop advanced treatment strategies for this disease and improve patient care.

Numerous studies have demonstrated the potential involvement of dysregulation of lncRNA expression in the pathogenesis of colon cancer and clinical significance. Previous studies have revealed that lncRNAs serve primarily as signaling molecules in a number of critical colon cancer-related pathways or as scaffolding guides for the induction of colon cancer-specific protein partners and as cis- and trans-regulatory elements of gene expression [21]. Some oncogenic lncRNAs promote colon cancer cell carcinogenesis, proliferation, invasion, metastasis, and drug resistance. For example, lncRNA SNHG11 promotes cell proliferation, migration, and invasion and is may be a novel therapeutic target for the treatment of colon cancer and a potential biomarker for early detection of colon cancer [22]. The lncRNA GLCC1 is an oncogene in colon cancer and is involved in cellular glycolysis [23]. Additionally, some lncRNAs have been found to inhibit the proliferation and metastasis of colon cancer cells. For instance, the lncRNA TUSC7 can inhibit epithelial-mesenchymal transition (EMT) in colon cancer cells, thus suppressing invasion and metastasis [24].

Our study found that PTOV1-AS2 was highly expressed in colon cancer tissues and cell lines and promoted proliferation, migration, and invasion of colon cancer cells. These findings imply that PTOV1-AS2 may have an oncogenic function in colon cancer. We also verified by survival curve analysis that PTOV1-AS2 expression has prognostic value for colon cancer patients, i.e., patients with high expression of PTOV1-AS2 showed worse prognosis. However, there are not many studies on PTOV1-AS2. Wang et al. developed a lncRNA prognostic model of head and neck squamous cell carcinoma, which included PTOV1-AS2 and found that its expression was significantly higher in cancer tissues than in normal tissues, and PTOV1-AS2 was found to be sensitive to four small molecule drugs (nelarabine, decitabine, acrichine, and dasatinib) [25]. Liu et al. identified seven genes (including PTOV1-AS2) that could be used as potential candidate biomarkers for predicting the prognosis of pancreatic cancer based on a novel TP53 correlation line graph that could predict the overall survival of pancreatic cancer patients using a risk score [26].

Dysregulated miRNA expression has a functional role in the progression and metastasis of colon cancer, acting as a tumor suppressor or oncogene to regulate the expression of its specific mRNA targets [27]. For example, miR-19a inhibits colon cancer angiogenesis by targeting KRAS and VEGFA [28], and miR-181d reduces cell proliferation, migration, and invasion by triggering PEAK1, a downstream regulator of the EGFR/KRAS pathway [29]. Several studies have indicated that lncRNAs are involved in the progression of a number of cancers by coordinating with miRNAs. A potential mechanism of lncRNAs in colorectal cancer is through binding to proteins to form complexes to regulate the expression of downstream miRNAs, by acting as miRNA sponges (ceRNAs) or scaffolds that regulate relevant signaling pathways and cellular processes [21]. For example, Liu et al. showed that lncRNA XIST promotes proliferation and EMT of colorectal cancer cells by targeting miR-486-5p and promoting neuropilin-2 [30]. Also, the lncRNA PAGBC acts as a microRNA sponge and promotes gallbladder tumorigenesis [31].

We predicted and confirmed that LncRNA PTOV1-AS2 binds to miR-145-5p and miR-145-5p binds to the FSCN1 3′-UTR region. Additionally, we showed that interfering with miR-145-5p or overexpressing FSCN1 could reverse the effect of silencing PTOV1-AS2 on the biological function of colorectal cancer cells. This finding was also verified by *in vivo* experiments in a nude mouse tumor model. Several studies have reported that miR-145-5p inhibits colon cancer, for example, miR-145-5p was found to inhibit colon cancer cell proliferation, metastasis, and EMT by targeting CDCA3 [32]. Also, miR-145-5p overexpression was shown to downregulate RHBDD1 by inhibiting EGFR-related signaling pathways (EGFR/Raf/MEK/ERK axis), thereby inhibiting colon cancer tumorigenesis [33]. However, to the best of our knowledge, no studies have been reported on the association of the binding of miR-145-5p to FSCN1 with colon cancer. Several reports have identified a useful prognostic value of FSCN1 in colon cancer, and targeting FSCN1 may provide potential therapeutic opportunities for colon cancer. For example, LINC00152 interacts with miR-632 and miR-185-3p as a ceRNA to regulate FSCN1 expression, thereby promoting malignant proliferation and metastasis [34]. This is consistent with the regulation of the expression level of FSCN1 in our study. There are also some limitations to this study. First, there are many potential binding miRNAs for the lncRNA PTOV1-AS2, and only miR-145-5p was selected for investigation in this study. Secondly, we did not perform a study targeting the downstream signaling of miR-145-5p/FSCN1. In addition, this study needs to further validate the function of PTOV1-AS2 in an *in situ* tumourigenic animal model.

## 5. Conclusion

In summary, we investigated a little reported lncRNA, namely, PTOV1-AS2, which promotes the colon cancer cell growth. The underlying mechanism of PTOV1-AS2 enhancing the proliferation of colon cancer was by “sponging” miR-145-5p to upregulate FSCN1. The lncRNA PTOV1-AS2/miR-145-5p/FSCN1 axis requires further study to fully exploit its potential value in the diagnosis, treatment, and prognosis of colon cancer.

## Data Availability

No underlying data was collected or produced in this study.
